# Home Sweet Home: Transforming Aging in Place Through the 5Ms

**DOI:** 10.1093/geroni/igaf122.990

**Published:** 2025-12-31

**Authors:** Beth Fields, Leslie Scheunemann

## Abstract

Aging in place remains a high-priority goal for older adults. Achieving this goal requires innovative solutions to address common problems of aging. This symposium will explore cutting-edge approaches and technologies that support aging in place, focusing on the 5Ms framework (What Matters, Medication, Mentation, Mobility, and Multicomplexity). Presenter 1 will introduce CAPABLE (Community Aging in Place—Advancing Better Living for Elders) program, which combines home visits by an occupational therapist, a nurse, and a handyperson to prioritize “What Matters” to the individual and to enhance “Mobility” through home modifications for aging in place. Presenter 2 will discuss medication management for older adults with Alzheimer’s disease and related dementias and multiple chronic conditions, highlighting strategies to reduce polypharmacy and its associated risks, emphasizing “Medication” and “Multicomplexity” in maintaining engagement in health-promoting daily activity. Presenter 3 will present 3D modeling for remote home assessments, enhancing “Mobility” and “Mentation.” Finally, Presenter 4 will share her work on a mobile app for home assessment that crosscuts the 5Ms, using augmented reality to evaluate and modify living environments, making them safer and more supportive for aging in place. This symposium will conclude with a discussion on integrating these innovative approaches and technologies into existing healthcare systems to promote aging in place, providing practical insights into applying the 5Ms framework to enhance the lives of older adults, ensuring they can live independently and with dignity in their own homes. Environmental Gerontology Interest Group Sponsored Symposium

